# Healthy children, healthy country: the use of governing instruments in shifting the policy paradigm

**DOI:** 10.1186/1743-8462-1-4

**Published:** 2004-11-18

**Authors:** Sandra G Leggat

**Affiliations:** 1 School of Public Health, La Trobe University, Bundoora, Australia

## Abstract

The evidence on early childhood strongly suggests the need to shift child health policy from the current focus on social welfare to a socio-ecologically based approach. This paper reviews three governing instruments, exhortation, expenditure and regulation, that have been used by governments in Australia and discusses the relative effectiveness of these approaches in shifting the child health policy paradigm.

## The evidence for healthy public policy for children

There can be no keener revelation of a society's soul than the way it treats its children [1].

Research evidence has demonstrated that the experiences of early childhood can have a profound lifelong impact on a child's health, wellbeing and competence [2]. The importance of the early years of life in influencing future outcomes, such as crime, obesity, heart disease, mental health problems and poor school outcomes has been identified and highlighted [3]. While there are many factors found to influence rising crime rates, various researchers have identified children with manifested behaviour disorders in early childhood [4], academic difficulties and non-engagement in schooling [5], and the quality of neighbourhood supervision and support as contributing factors to criminal behaviour [6].

Education, literacy and other social determinants of health can influence the coping skills of children, which provide the basis of learning, behaviour and health throughout life [7]. Poverty, whether measured in absolute or relative terms, has a negative effect on children's health [8,9]. In particular, poverty is associated with developmental delay, poor school achievement and employment futures, behaviour problems, increased incidence of chronic illness, visual and hearing defects and dental problems [10]. Parental poverty and exposure to unhealthy environments (eg smoking; low levels of literacy; nutrition; emotional support) reduce a child's life chances. Studies in neurobiology, neurodevelopment and early intervention show that the time period from conception to school age is a critically important time for brain development, setting the scene for prevention of some of the identified adverse outcomes through early identification and intervention [11].

Consistent with the increasing evidence, many governments have identified support in early childhood as a lifelong determinant of health, wellbeing and competence, as a matter for policy development, initiating actions to ensure comprehensive child development strategies for their societies. This approach requires a whole of government response, integrating health, welfare, education and other relevant parts of government. The evidence suggests that healthy public policy for infants, children and their parents is dependent on understanding of the socio-ecological factors supported by integrated, multidisciplinary and intersectoral policy and programs.

In Canada, Britain and the United States, targeted interventions in the antenatal period, infancy and childhood, including parenting skills programs, are recognized for their potential to support healthier families. A socio-ecological model of health is increasingly perceived to be the most appropriate approach for the early years of life agenda. Consistent with this approach, the United Kingdom program *'Sure Start'*, has been positively reviewed by the UK Audit Office and is considered by many to be the standard for the whole of government approach [12]. In addition, a recently released ten year plan is attempting to significantly change the way in which children are treated throughout UK systems [13].

The Canadian experience is widely quoted as best practice [2], with both federal and provincial investment in early childhood (See, for example [14-16]). In the US, during the Presidents' Summit for America's Future held in April 1997, Presidents Bill Clinton, George Bush, Jimmy Carter and Gerald Ford and First Lady Nancy Reagan stressed the importance of early childhood, calling the nation to action. American policy in this area has built upon influential reports that have led to investment in early childhood in most states [12].

The adoption of healthy public policy for children based on this socio-ecological framework has been inconsistent throughout Australia. In an attempt to explore these inconsistencies, this paper reviews the use of three governing instruments, that is exhortation, expenditure and regulation, by national and state governments in Australia. Governing instruments are the major mechanisms governments use to seek compliance, support and implementation of public policy. Governing instruments range from minimum coercion by exhortation, through expenditure, taxation, regulation, to maximum coercion through public ownership [17]. The following sections describe the impact of the use of exhortation, expenditure and regulation on the implementation of healthy child policy.

## Consensus building – exhortation as the national instrument of choice

During the 1990s the Australian Government identified the health of children and young people as a key policy area, with a series of policy documents:

• *The National Health Goals and Targets for Australian Children and Youth *(1992)

• *The National Health Policy for Children and Young People *(1995) and associated *Implementation Plan *(1996)

• *The National Health Policy for Young Australians *(1997).

These documents provided broad national goals for children and young people:

• Reducing preventable premature mortality

• Reducing the impact of disability

• Reducing the incidence of vaccine preventable disease

• Reducing the impact of conditions occurring in adulthood with their origins or early manifestation in childhood or adolescence

• Enhancing family and social functioning

Although the evidence supporting a broader definition of child health was strong, the focus of these National Health Goals and Targets remained heavily focused towards surveillance and the reduction of injury and illness, perhaps reflecting a comfort with current and past approaches.

To date there has been little evidence of an integrated, multidisciplinary approach to child health at the national level. The 2003/04 federal budget did not provide the broad whole of government approach recommended for child health, with only a few targeted interventions, such as the National Meningococcal C Campaign, and a much greater focus on the health needs of the aging population. In 2003, the Australian of the year, Fiona Stanley suggested that the social and economic policies of the Government were not effective in tackling the issues associated with ensuring healthy children and young people [18].

The platform for a paradigm shift was established in 2001 with the appointment of the Minister for Children and Youth Affairs and the subsequent statement in 2002 of the intent to develop a National Agenda for Early Childhood. The consultation paper *Towards the Development of a National Agenda for Early Childhood *signaled a changing paradigm, with a whole of child and life course approach addressing promotion, prevention and early intervention for all children.

The last years have seen the creation of ever more advisory groups, partnerships and inquiries with a mandate to influence child health policy. The Child and Youth Health Intergovernmental Partnership (under the auspice of the Australian Health Ministers' Advisory Council) was convened in December 2001 to develop a national child public health strategy and advise on the National Agenda for Early Childhood. Their draft strategy framework *Better Child Public Health: A Strategic Approach to Building Capacity – A National Action Plan 2004–2007 *has been developed and is being used in consultation and capacity building initiatives. In October 2002 the Minister for Children and Youth Affairs referred an inquiry into improving children's health and well being to the Standing Committee on Family and Community Affairs. The Australian Council for Children and Parenting (ACCAP), an advisory body to the Minister for Children and Youth Affairs, was granted a two year term from July 2003, with a focus on strategic advice in the areas of early childhood intervention and prevention, parenting and child protection, foster care and emerging early childhood initiatives, including advising about the continuing development of the National Agenda for Early Childhood.

As described above, the policy approach at the national level has focused almost entirely on exhortation, the least coercive instrument, where support and compliance are sought voluntarily through persuasion and discussion. In comparison with other countries, such as Britain and Canada, the lack of a common and shared understanding of the socio-ecologic approach and its implications has made it difficult to show any significant advances in this area. In fact, the recent demise of the Child Health Unit within the Australian Government Department of Health suggests less focus on child health.

Nationally, child health has not been heavily addressed through other policy instruments, such as expenditure, taxation, or regulation, although more recently the Commonwealth Government Department of Family and Community Services (DFaCS) established the *'Communities for Children*' initiative as part of the Stronger Families and Communities Strategy. *Communities for Children *will directly fund 35 Australian communities between $1 and 4 million over four years to support parents, neighbourhoods and the wider community to give children the healthy start they need [19]. Importantly, there was little evidence of a community development or even a consultative approach in the implementation of this program, with the perception that *Communities for Children *has not been set up to respond to the greatest need.

It has been suggested that system change can be accomplished by motivating institutions, systems and actors to move in common directions and develop structures that sustain these efforts over time [20]. This requires a high level of trust among the participants, such that they eventually share common goals and voluntarily seek to achieve common ends. Success in using exhortation as a policy instrument requires that information not only flow from government, but also to it [21]. The strong use of exhortation at the national level may be seen as the only way to encourage change, given the shared responsibility for child welfare among the various levels of government in Australia. Yet it is precisely this divided accountability and responsibility that has been identified 'as the greatest barrier to the reform of children's services' [[22] pg. 980].

The use of exhortation may be successful at motivating common approaches but will be much less effective at ensuring the sustaining structures are developed. This is apparent in the existing committee structures, which still operate from within the government structures and are thus unable to cross the 'silos' to promote the needed whole-of-government approach. To be effective in changing the paradigm in this area, the exhortation process will require back up by more coercive governing instruments.

## Conflicting expenditures – potential for uncertain outcomes in Victoria

In comparison with other Australian states, Victoria has been slow to provide visible translation of the socio-ecological model of health for children and young people in a coordinated and systematic way to state policy. A recent review of Victorian paediatric services suggested that Victoria needed to establish a child and young people focus ensuring appropriate mechanisms to plan, coordinate and monitor across government departments and service providers [23]. It was suggested that a structure was required to coordinate child health among of the various portfolios in the Victorian Department of Human Services – Health, Housing, Welfare, and Disability – as well as among the broader Government departments, contributing to the whole of government approach required for early childhood intervention programs.

The lack of coordinated focus on child health in Victoria is perhaps the result of a lingering policy focus on health surveillance. Despite the increasing evidence that surveillance and screening programs have limited effectiveness in child health [24,25], it is only recently that Victoria has increased the focus on the social determinants of health [26,27]. Most recently, Victoria has committed to the *'Best Start' *program and will pilot it as demonstration projects in 10 communities across the State with an investment of $7.6 million. *Best Start *is auspiced by the Departments of Human Services and Education and Training and is focused on reducing the impact of disadvantage (from any cause) and enhancing the life chances of all children by strengthening the universal preventative system [28]. The aims of *Best Start *are multi-level including the social, emotional and physical well-being of children, capacity building of parents and carers and communities to assist them to become more child friendly, while focusing on specific interventions for socially disadvantaged families [29]. The demonstration projects are required to follow a prescribed implementation and evaluation process attempting to measure what works, under what circumstances and for whom, to ultimately improve services elsewhere in the State. Five approved demonstration sites with a total of $7.6 million are ensuring a 'brighter future' for the children of Frankston, Hume, Shepparton, Whittlesea and Yarra Ranges, while the rest of the State's children wait in the dark.

Despite the intentions of *Best Start*, existing government funding and reporting in the area of maternal and child health is still largely focused on surveillance [26]. The Victorian approach to policy implementation in the area of early childhood support is focused predominantly on expenditure. Public expenditure is moderately coercive, with distribution of government funds to achieve particular policy objectives. But the small expenditure allocated to 'healthy' child policy that is limited to identified demonstration sites with expectations that the program will be shown to be effective before statewide mainstream implementation is overshadowed by a much larger expenditure pool that is not focused on the socio-ecological model. While *Best Start *signals intent to change the child health policy paradigm, the incentives established through the broader expenditure pool suggest, for the moment, maintenance of the status quo in Victoria.

## Guidelines – will enforcement back the regulatory approach in NSW?

In New South Wales the *'Families First' *initiative targets families with children 0 to 8 years, with the aim of helping parents give their children a good start in life. Demonstrating the commitment to a whole of government approach, the Office of Children and Young People (OCYP), located within The Cabinet Office, reporting directly to the Premier, has played a lead role in the development and implementation of the *Families First *strategy. This evidence-based approach is delivered jointly by five NSW government agencies – Area Health Services, Community Services, Education and Training, Housing and Disability, Ageing and Home Care in partnership with parents, community organisations and local government. NSW Health supports 'the ongoing development of partnerships at policy, planning and service delivery levels to enable improved co-ordination and intersectoral collaboration in the delivery of child health services' [[30] pg. 44].

NSW has also successfully translated much of the evidence into coordinated service planning and delivery at the regional level. Paediatric networks, associated with the Area Health Services, were established in 1997 and today provide designated primary, secondary and tertiary level services for families with children aged 0 to 5 years [31]. In addition, the NSW Commission for Children and Young People focuses on increasing the participation of children and young people in decision making that affects their lives, promoting the safety and welfare of children and young people, and strengthening the important relationships in the lives of children and young people and improving their well-being [32].

The implementation of *Families First *has been guided by a series of policy and practice guidelines. Recently, an independent review of *Families First *implementation within three regions, (Orana Far West, Illawarra and South West Sydney) found that the system changes required to build and strengthen service networks for families needed more than agreement and goodwill, with considerable effort to develop structures and processes that sustain interagency collaboration [33]. This resulted in a further guide to implementing sustainable and effective child and family service networks.

Regulation involves the imposition of requirements to meet specific obligations. Often regulation is seen to exist within legislation outlining strict rules of behaviour. However, in health policy guidelines are considered effective means of imposing regulation, recognising the inherent uncertainty in safe practice in health care [17]. The implementation of 'healthy' child policy in NSW suggests a strong focus on the socio-ecological approach supported by the research evidence. This is apparent in the whole of government approach with leadership from the Premier's Office, backed by regulation to effect the necessary changes in the delivery system.

However, it is yet to be seen whether the policy will be supported with the necessary resources for compliance. While regulation involves shifting costs of compliance from government to other participants, enforcement and monitoring can be expensive and difficult [21]. Without adequate enforcement the potential for inequality and inequity in access to the proposed service model is high.

## Conclusions

A variety of instruments have been used by government to change the child health policy paradigm from that focused on social welfare to 'healthy' public policy predicated on a socio-ecological foundation. NSW has chosen regulation to implement a child health policy framework that is built upon the international evidence of the effectiveness of integrated, multidisciplinary and intersectoral policy and programs. A little slower to change paradigms, Victoria has established demonstration programs through targeted expenditure, without an overarching whole-of-government child health policy framework. Nationally, there is a move to change the paradigm to a broader definition of child health almost exclusively through exhortation.

Instrument choice is influenced by a variety of factors. The use of exhortation by the Commonwealth Government is a relatively risk-free easy approach, which can counteract the divided accountabilities among federal and state governments in the area of health and social services. Exhortation is easy to implement; it is the least coercive and relies on voluntary goodwill. While it may be successful in building a common understanding, and even this is debatable, an independent review of the *Families First *implementation found that the system changes required to build and strengthen service networks for families needed more than agreement and goodwill to develop the necessary structures and processes – a suggestion that without other approaches to structural reform, exhortation is unlikely to be successful.

The expenditure policy of Victoria illustrates an approach that is compromised by the lack of an underlying agreed evidence-based policy framework. This lack of coordinated government approach is reflected in conflicting expenditure policy in this area, with the potential to confound outcomes.

While the regulatory approach of NSW suggests bold steps to change the paradigm, in fact, because regulation is not subjected to the same level of scrutiny of other instruments, such as expenditure, and even exhortation [17], it is a deceptively simple mechanism to implement policy [21]. The strength of the government intent to change the paradigm will only become apparent with visible enforcing of the service delivery directions.

The evidence for a new policy paradigm is strong. But the use of these different policy instruments underscores the lack of shared understanding and policy agenda. Oberklaid suggests that while there are similarities in the rhetoric throughout Australia, there has been relatively little investment in child health [12]. The change in the child health public policy paradigm will only be successful when the governing instrument or combination of instruments induces the appropriate public and private behaviour. Perhaps we should be thankful that child health policy is on the agenda, and even without a strong, coordinated approach built on the evidence, one would agree that 'these developments in early childhood services demonstrate the translation of research evidence into policy and practice, even if the implementation may be flawed, belated or under-resourced' [[5] pg.15].

## Competing interests

The authors declare that they have no competing interests.
